# Nasal Mucosal Necrosis in a Patient with Systemic Lupus Erythematosus and Antiphospholipid Syndrome: A Case Report and Literature Review

**DOI:** 10.3390/jcm15145711

**Published:** 2026-07-21

**Authors:** Dong Hoon Lee, Ki-Jeong Park, Seo-Yeon Ahn, Joo Yeon Koo, Sang Chul Lim

**Affiliations:** 1 Department of Otolaryngology-Head and Neck Surgery, Chonnam National University Medical School, Chonnam National University Hwasun Hospital, Hwasun 58128, Republic of Korea; 2 Department of Rheumatology, Chonnam National University Medical School, Chonnam National University Hospital, Gwangju 61469, Republic of Korea; 3 Department of Hematology-Oncology, Chonnam National University Medical School, Chonnam National University Hwasun Hospital, Hwasun 58128, Republic of Korea; 4 Department of Pathology, Chonnam National University Medical School, Chonnam National University Hwasun Hospital, Hwasun 58128, Republic of Korea

**Keywords:** antiphospholipid syndrome, systemic lupus erythematosus, invasive fungal sinusitis, thrombocytopenia, thrombopoietin receptor agonists, microthrombi

## Abstract

**Background:** The clinical presentation of nasal mucosal necrosis, an exceedingly rare manifestation of secondary antiphospholipid syndrome (APS) in patients with systemic lupus erythematosus, often mimics invasive fungal sinusitis (IFS), causing diagnostic challenges and potentially unnecessary surgical interventions. Here, we report a unique case of nasal necrosis exacerbation by thrombopoietin receptor agonist (TPO-RA) therapy. **Case Description:** A 54-year-old woman with systemic lupus erythematosus and APS developed rapid nasal mucosal necrosis after receiving TPO-RA. An initial clinical suspicion of IFS led to emergency surgical debridement, and histopathology revealed fibrin-rich intraluminal microthrombi without fungal elements. After replacing eltrombopag with fostamatinib (a Syk inhibitor), the nasal lesions improved markedly, with complete mucosal healing. **Conclusions:** Nasal mucosal necrosis, a rare but serious symptom of secondary APS, requires careful differential diagnosis from IFS or other necrotizing diseases. APS-related vasculopathy should be considered following TPO-RA therapy, particularly if repeat debridement fails to identify an infectious cause.

## 1. Introduction

Antiphospholipid syndrome (APS) is an immune-mediated or autoimmune thromboinflammatory disorder characterized by recurrent arterial or venous thrombosis, pregnancy morbidity, and the persistent presence of antiphospholipid antibodies [1,2,3,4,5,6,7]. APS pathogenesis is driven by complex interactions between antiphospholipid antibodies, particularly anti-β2-glycoprotein I (anti-β2GPI), which is considered a key pathogenic antibody, and hemostatic and vascular cells [1,2,3]. These antibodies directly stimulate platelets by increasing glycoprotein IIb/IIIa expression and thromboxane A2 production, thereby enhancing aggregation and clot formation [1,5]. This underlying prothrombotic state acts as a “first hit”. Often, a “second hit”—an additional trigger such as infection, surgery, hormonal changes, or certain medications—is necessary to precipitate an acute thrombotic event [3,5]. This concept has relevance when considering iatrogenic risk factors in APS patients.

While most thromboembolic events in APS manifest as deep vein thrombosis, pulmonary embolism, or stroke, sinonasal ischemic necrosis is uncommon and may be under-recognized in clinical practice [1,2,3]. The rarity of this diagnosis has important practical consequences because in immunologically complex patients, necrotic sinonasal lesions often raise concerns of invasive fungal sinusitis (IFS), a surgical and infectious emergency [1,2,3]. Distinguishing APS-related ischemic necrosis from IFS has major practical significance. IFS often prompts aggressive debridement because delayed intervention can be fatal, particularly in immunocompromised hosts. However, unnecessary radical sinonasal surgery in a patient whose lesion is ischemic rather than infectious may worsen bleeding risk, delay initiation of targeted systemic therapy, and add morbidity without therapeutic benefit.

Thrombopoietin receptor agonists (TPO-RAs), such as romiplostim and eltrombopag, are effective second-line treatments for refractory immune thrombocytopenia (ITP) [8,9]. These agents increase platelet production via activation of JAK/STAT and MAPK signaling pathways. However, these agents carry a paradoxical thrombotic risk beyond simple platelet count elevation. A recent French multicenter study highlighted the significant thrombotic risk of TPO-RAs in patients with ITP secondary to systemic lupus erythematosus (SLE) and APS [4], aligning with the APS management algorithm that emphasizes personalized risk stratification before using prothrombotic agents [7]. In addition, cardiovascular and clinical risk factors have important prognostic and therapeutic value in patients with SLE and APS and should be incorporated into treatment planning and surveillance [8]. Here, we describe a case of nasal mucosal necrosis associated with APS following TPO-RA therapy, which improved after TPO-RA withdrawal. This case underscores the thrombotic hazards of TPO-RAs in APS, the diagnostic challenge of differentiating thrombotic vasculopathy from invasive infection, and the critical role of multidisciplinary management.

## 2. Case Report

A 54-year-old female with a history of SLE, hypertension, and refractory thrombocytopenia was referred to the emergency room in late July 2025 because of rapid-onset left periorbital swelling and severe facial pain. The patient had been diagnosed with secondary APS associated with SLE in 2001 and managed for recurrent abortion and thrombocytopenia without thrombotic events. The diagnosis met both the historical (1997 ACR for SLE; 1999 Sapporo for APS) and contemporary classification criteria (2019 ACR/EULAR for SLE; 2023 ACR/EULAR for APS). Her antiphospholipid antibody profile had been persistently positive since May 2015, demonstrating a strongly positive lupus anticoagulant (ratio > 2.3), elevated anti-cardiolipin IgG (>120 U/mL; IgM negative), and elevated anti-β2-glycoprotein I IgG (>50 U/mL; IgM negative). Her maintenance medications included hydroxychloroquine (200 mg) for SLE, as well as valsartan (80 mg) and amlodipine (5 mg) for hypertension.

**Figure 1:**
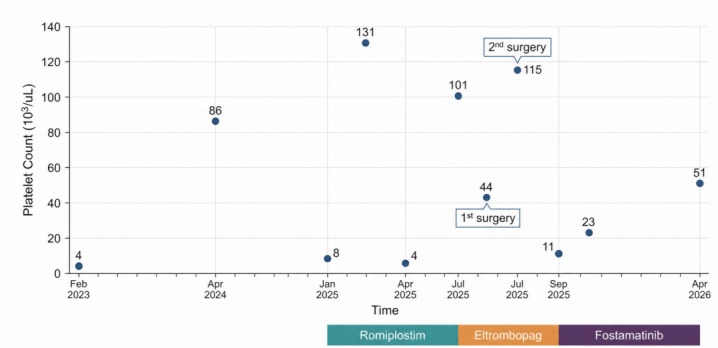
Platelet count trajectory and clinical intervention.

Her thrombocytopenia worsened significantly beginning in February 2023, with platelet counts frequently dropping below 10,000/μL. Initial management consisted of high-dose intravenous methylprednisolone and intravenous immunoglobulin (IVIG). However, she exhibited severe steroid dependence; attempts to taper prednisolone below 20 mg/day consistently resulted in a rapid platelet count decline under 10,000/μL (Figure 1). Mycophenolate mofetil was introduced as a steroid-sparing immunosuppressant but was discontinued due to severe gastrointestinal intolerance and patient refusal. Rituximab, although considered a preferred second-line option for refractory SLE-associated ITP, was not readily accessible due to strict national health insurance reimbursement restrictions in South Korea.

Given the refractory nature of her thrombocytopenia, romiplostim (250 mcg weekly) was initiated in January 2025. Although her platelet counts successfully increased to over 100,000/μL, romiplostim was discontinued after two months due to intolerance. In April 2025, treatment was switched to oral eltrombopag (50 mg daily), which successfully maintained her platelet counts above 50,000/μL until her presentation to the emergency room (Figure 1).

**Figure 2:**
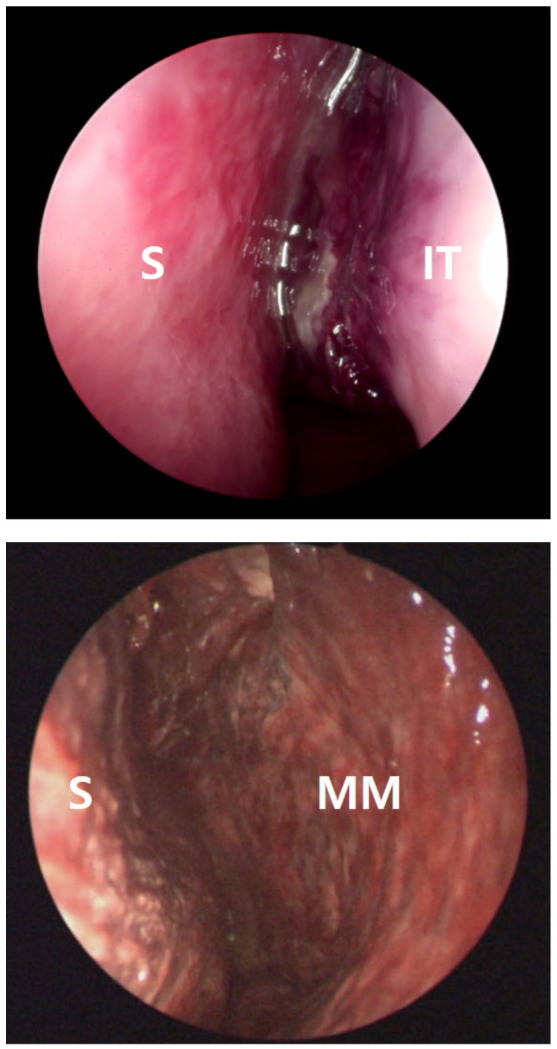
Nasal endoscopy shows a necrotic appearance of the left inferior turbinate (A, IT) and middle meatus (B, MM). S—septum; IT—inferior turbinate; MM—middle meatus.

Nasal endoscopy at the emergency room revealed a necrotic appearance of the left inferior turbinate and middle meatus (Figure 2). Given the patient’s immunocompromised state resulting from SLE and systemic corticosteroid therapy, the sudden onset of nasal mucosal necrosis immediately raised a high suspicion for IFS. Given the clinical urgency, the patient immediately underwent emergency endoscopic sinus surgery and debridement on 21 July 2025.

**Figure 3:**
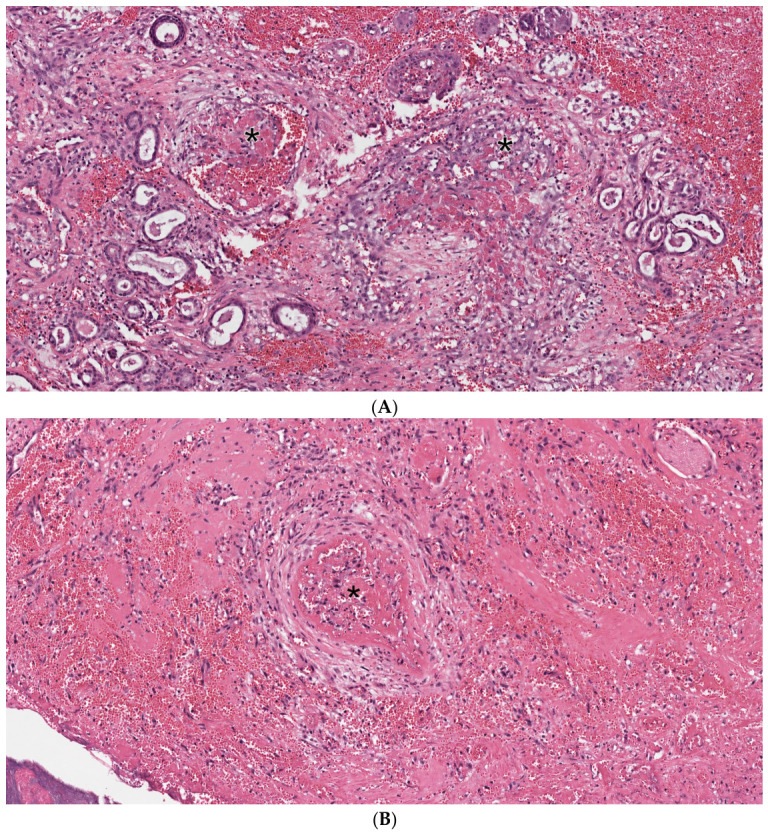
Histopathologic findings of the left inferior turbinate. (**A**) Sinonasal mucosa showing thrombotic occlusion of small vessels with associated stromal hemorrhage and ischemic tissue injury. Asterisks indicate intraluminal fibrin-rich thrombi in affected vessels. (**B**) A small vessel exhibits marked concentric wall thickening and luminal obliteration by fibrinous thrombotic material, consistent with obliterative thrombotic vasculopathy (asterisk). Hematoxylin and eosin staining (original magnification: 100×).

Histopathologic examination of sinonasal biopsies revealed acute fibrinoid necrosis with intraluminal fibrin-rich thrombi (microthrombi), and there was no evidence of fungal hyphae or bacterial colonization (Figure 3). Fungal cultures were negative, and periodic acid–Schiff (PAS) and Grocott methenamine silver (GMS) stains also showed no fungal organisms. Serologic evaluation for AN-CA-associated vasculitis was negative, and the histologic findings did not support vasculitis, helping to exclude ANCA-associated vasculitis from the differential diagnosis. The necrotic area did not show evidence of fungal infection or other diseases during the second debridement on 29 July 2025. These findings indicated the presence of thrombotic vasculopathy, rather than IFS.

**Figure 4:**
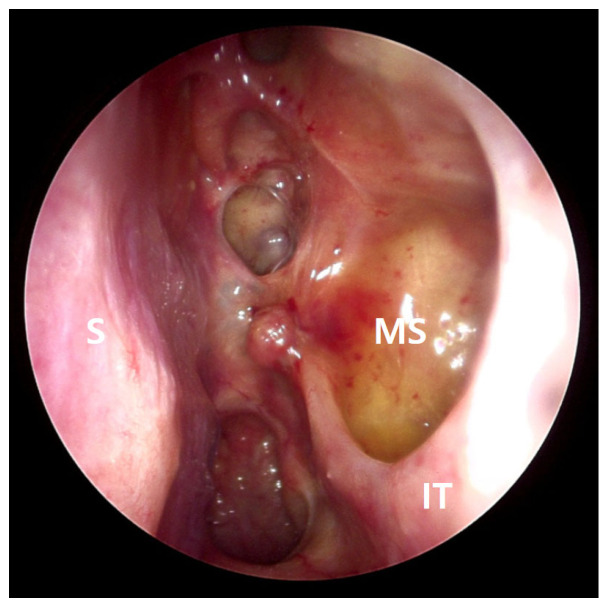
Nasal endoscopy eight months after surgery revealed complete nasal cavity re-epithelialization with a normal clinical appearance. S—septum; IT—inferior turbinate; MS—maxillary sinus.

Following a multidisciplinary consultation, nasal necrosis was considered a manifestation of secondary APS during TPO-RA use. Eltrombopag was discontinued after its identification as a potential contributor to thrombotic vasculopathy. The patient’s SLE and APS stabilized with intensified management, and treatment for ITP was changed to fostamatinib (a Syk inhibitor) on 22 September 2025, which successfully maintained her platelet counts at approximately 20,000/μL or higher (Figure 1). She was subsequently managed long term with anticoagulation using warfarin, with a target international normalized ratio (INR) of 2.0–3.0, together with ongoing rheumatologic and hematologic follow-up. Following this change and the stabilization of SLE and APS, the patient’s nasal mucosa began to improve dramatically in September 2025. Nasal endoscopy eight months after surgery revealed complete nasal cavity re-epithelialization, with a normal clinical appearance (Figure 4).

## 3. Discussion

In patients with autoimmune diseases, necrotic sinonasal lesions present a major diagnostic challenge because secondary APS involving the nasal mucosa is rare, and its gross appearance and clinical urgency can closely mimic IFS [1,4,5,6,7]. Moreover, misdiagnosis may expose patients to unnecessary repeat debridement, prolonged empiric antifungal therapy, and delayed recognition of the true vascular process. Tissue diagnosis was decisive in the present case, with fibrinoid necrosis with intraluminal fibrin-rich microthrombi being the histopathologic hallmark, while fungal elements were absent based on routine histology, fungal culture, PAS stain, and GMS stain [1,2,3]. Because the patient was immunocompromised and had a black necrotic eschar on nasal endoscopy, the initial suspicion of IFS was clinically appropriate. However, the pathologic findings redirected management away from persistent concerns about IFS and toward APS-related thrombotic vasculopathy [1,2,3,4,5,6,7]. ANCA-associated vasculitis was also considered, but negative serology and the absence of histologic vasculitis argued strongly against that diagnosis.

The potential contribution of TPO-RAs to thrombotic vasculopathy in patients with APS is the most important point in this case [4,5,8,9]. Although TPO-RAs, e.g., eltrombopag, are effective agents for thrombocytopenic disorders, they carry a thrombotic risk. By elevating platelet counts and enhancing prothrombotic signaling, TPO-RAs can precipitate microthrombi in susceptible tissues [4,5,8,9]. Furthermore, it is important to recognize that thromboembolic episodes in patients with SLE are often driven by a complex interplay of cumulative cardiovascular and clinical risk factors. Recent data suggest that individualized thrombotic risk assessment is crucial, as the coexistence of traditional cardiovascular risks and SLE-specific activity markers significantly elevates the overall vascular risk [10,11]. In a patient already predisposed to thrombosis by SLE and APS, eltrombopag likely acted as a critical “second hit,” triggering microvascular occlusion in the nasal mucosa. The temporal improvement observed after TPO-RA withdrawal further supports its role in the thrombotic process [7]. Recent clinical data strongly support this concern. The French multicenter study by Marques et al. demonstrated a 50% thrombotic event rate in APS patients treated with TPO-RAs, compared to 8.1% in SLE patients without antiphospholipid antibodies [4]. Notably, five of the eight APS patients who developed thrombotic events had no prior thrombotic history, and the median time to thrombosis was approximately 100 days after TPO-RA initiation [4]. Considering these clinical data and our present findings, TPO-RAs should be used with extreme caution—or avoided entirely—in patients with definite APS. Smaller case reports and series also suggest that TPO-RAs may unmask or amplify thrombotic susceptibility in APS, particularly when additional risk factors such as active systemic inflammation, hypertension, immobilization, or other cardiovascular risk factors are present. These observations support a cautious, individualized approach to TPO-RA use in APS and reinforce the need for close clinical surveillance [1,2,3].

Equally importantly, this case illustrates the value of tissue diagnosis before committing to destructive surgery [1,2,3,4,5,7]. Because IFS requires urgent debridement and antifungal therapy, the threshold to intervene is understandably low when necrotic tissue is seen [1,2,3]. When pathology excludes fungal invasion and the broader clinical context favors ischemic APS-related injury, conservative management with targeted medical therapy may spare the patient from unnecessary aggressive surgery and its associated morbidity.

The broader implication is that sinonasal necrosis should be approached as a multidisciplinary diagnostic emergency rather than an automatic indication for radical debridement. Otolaryngology, hematology, infectious disease, pathology, and radiology input may be required to rapidly separate invasive infection from sterile thrombotic necrosis. Recognition of this distinction can directly alter treatment intensity, timing of systemic therapy, and ultimate outcomes.

In this case, thrombocytopenia was managed using a SyK inhibitor, which, unlike TPO-RAs, targets the Fc-gamma receptor signaling pathway in macrophages, thereby reducing platelet destruction without directly stimulating prothrombotic pathways [7]. By modulating Fc-gamma receptor-dependent signaling, fostamatinib may reduce antibody-mediated platelet activation, macrophage activation, and amplification of thromboinflammatory cascades. This mechanism is particularly relevant in disorders where autoantibody-driven cellular activation and immunothrombosis intersect. In APS biology, antiphospholipid antibodies can promote endothelial injury, monocyte tissue factor expression, platelet activation, neutrophil extracellular trap formation, and complement activation. Rapid nasal mucosa re-epithelialization was observed upon TPO-RA withdrawal and transition to a SyK inhibitor. This finding, combined with stabilization of SLE and APS, supports the hypothesis that removing the thrombotic trigger while addressing the underlying autoimmune process allowed vascular healing.

## 4. Conclusions

Nasal mucosal necrosis, a rare but serious symptom of secondary APS, requires careful differential diagnosis from IFS or other necrotizing diseases. Clinicians should include APS-related thrombotic vasculopathy in the differential diagnosis of necrotic sinonasal lesions, pursue timely biopsy with careful histopathologic evaluation, and review medication history for the use of prothrombotic agents. Awareness that TPO-RAs may contribute to thrombotic vasculopathy, combined with a multidisciplinary approach, may facilitate timely recognition and appropriate management of patients with APS presenting with nasal mucosal necrosis.

## Data Availability

The data presented in this study are available on request from the corresponding author.
